# Effect of Incorporating Polyvinyl Alcohol Fiber on the Mechanical Properties of EICP-Treated Sand

**DOI:** 10.3390/ma14112765

**Published:** 2021-05-23

**Authors:** Hua Yuan, Guanzhou Ren, Kang Liu, Zhiliang Zhao

**Affiliations:** 1 School of Civil Engineering and Architecture, Henan University, Kaifeng 475004, China; yuanhua@henu.edu.cn (H.Y.); 104754180900@vip.henu.edu.cn (G.R.); 104754190839@henu.edu.cn (K.L.); 2 Henan Rail Transit Intelligent Construction Engineering Research Center, Kaifeng 475004, China; 3 Central Plains Development Research Institute, Henan University, Kaifeng 475004, China

**Keywords:** enzyme-induced calcium carbonate precipitation, polyvinyl alcohol fiber, sand, unconfined compressive strength, calcium carbonate content, ductility

## Abstract

Enzyme-induced calcium carbonate precipitation (EICP) technology can improve the strength of treated soil. But it also leads to remarkable brittleness of the soil. This study used polyvinyl alcohol (PVA) fiber combined with EICP to solidify sand. Through the unconfined compressive strength (UCS) test, the effect of PVA fiber incorporation on the mechanical properties of EICP-solidified sand was investigated; the distribution of CaCO_3_ in the sample and the microstructure of fiber-reinforced EICP-treated sand were explored through the calcium carbonate content (CCC) test and microscopic experiment. Compared with the sand treated by EICP, the strength and stiffness of the sand reinforced by the fiber combined with EICP were greatly improved, and the ductility was also improved to a certain extent. However, the increase of CCC was extremely weak, and the inhomogeneity of CaCO_3_ distribution was enlarged; the influence of fiber length on the UCS and CCC of the treated sand was greater than that of the fiber content. The improvement of EICP-solidified sand by PVA fiber was mainly due to the formation of a “fiber–CaCO_3_–sand” spatial structure system through fiber bridging, not the increase of CCC.

## 1. Introduction

Microbial-induced calcium carbonate precipitation (MICP) [1] and enzyme-induced calcium carbonate precipitation (EICP) are novel environmentally friendly biogrouting technologies in which EICP technology uses urease directly to induce CaCO_3_ precipitation, thus eliminating the tedious microbial culture [2,3]. Both technologies are widely used in soil reinforcement [4,5,6]. Previous scholars [7,8] have successfully used PVA fiber to improve cement-based composites. In order to compensate for the undesirable weakness of the brittle failure and low residual strength of biocemented soil, researchers have attempted to combine MICP technology with fiber reinforcement. Li et al. [9] used a full-contact flexible mold to prepare MICP-treated sand samples with added fiber. Their results showed that fiber cooperation can improve shear strength and failure strain. Xie et al. [10] found that adding fiber to reinforced microbial sand can greatly enhance the unconfined compressive strength (UCS) of soil samples and significantly improve the samples’ ductility. Akay [11] reinforced the sandy slope through MICP combined with fiber and found that the stability of the slope was improved and the development of failure surface and cracks were limited [12,13,14]. However, all of the above studies found that the use of MICP and fiber to improve soil is not conducive to the uniformity of soil reinforcement. The urease in EICP is free and lacks nucleation sites. Therefore, the mechanism of fiber-reinforced EICP may be very different from that of fiber-reinforced MICP, and it had not been found that scholars have carried out this study.

In order to study the effect of EICP and fiber-reinforced technology on soil treatment, polyvinyl alcohol (PVA) fibers at contents of 0.2, 0.4, and 0.6% by weight and at lengths of 3, 6, 9, and 12 mm were used together with EICP to treat Chinese standard sand. The influence of fiber on the mechanical properties and ductility of EICP-treated sand were investigated using the UCS and calcium carbonate content (CCC) tests. The microstructure of solidified soil samples was observed via microscope and scanning electron microscopy (SEM) to explore the action mechanism of the combination of fiber and EICP for soil improvement.

## 2. Materials and Methods

### 2.1. Materials

Considering that PVA fiber has fine resistance to acid, alkali, salt, wear, sunlight, and corrosion, the test used high-strength and high elastic modulus PVA fiber [15,16]. The test also adopted standard silica sand from Xiamen, China, whose particle distribution is shown in Table 1. The basic characteristic parameters of the sand and PVA fiber are shown in Table 2; the non-uniformity coefficient *C*_u_ is greater than 5, and the curvature coefficient *C*_c_ is between 1 and 3, indicating that the sand has good grain gradation.

The EICP treatment solution consisted of 100 g/L soybean urease and CaCl_2_–urea mixture ([CaCl_2_:urea] = 2:3, [CaCl_2_] = 1.0 M) [17,18]. The extraction of soybean urease [19] was as follows: the soybean was baked at 40 °C for 6 h and then crushed with a grinder and sifted through a 0.15 mm sieve. The soybean powder and deionized water were mixed in a beaker at a concentration of 100 g/L. The mixed solution was stirred electromagnetically for 30 min and centrifuged at 3000 r/min for another 30 min, and the bean dregs were removed. The upper supernatant of the remaining liquid was the urease solution after remaining in the refrigerator overnight.

### 2.2. Specimen Preparation

The mold used was a cylindrical acrylic pipe with an inner diameter of 39.1 mm and a height of 100 mm, which consisted of the splicing of two half molds. A permeable stone with a thickness of 10 mm was placed at the lower end of the mold. The prepared sand sample was a cylinder 39.1 mm in diameter and 80 mm in height. A layer of PVC film was used to separate the mold from the sand column, which was convenient for demolding after molding.

**Figure 1:**
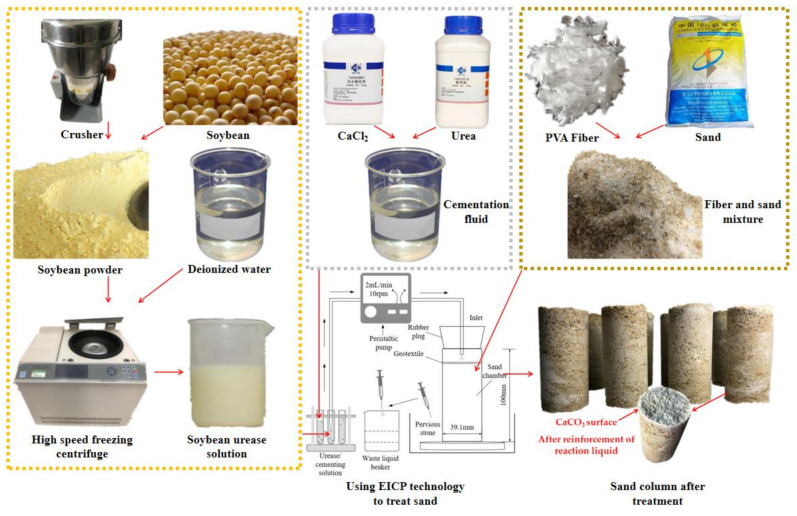
Schematic diagram of sample preparation.

The soil samples were prepared as follows: (1) 161.53 g (calculated based on the initial parameters) of standard sand and a certain amount of fiber were weighed. The two were mixed evenly, divided into three equal parts, and poured into the mold successively. After each pouring, they were compacted to a predetermined height. (2) Fifty milliliters of soybean urease solution was injected from the top of the soil sample at a rate of 2 mL/min using a peristaltic pump. The mixture rested for 5 h to ensure that the urease was fully diffused in the soil sample and adsorbed on the surface of the sand particles. (3) Fifty milliliters of the CaCl_2_–urea mixture was injected into the soil sample in the same way, and the mixture was left to stand for 24 h, which was recorded as a cycle. (4) After seven cycles, the soil sample was solidified. The sand treatment process by EICP and the prepared soil samples are shown in Figure 1.

### 2.3. Tests and Methods

The PVA fibers at dosages of 0.2, 0.4, and 0.6% by sand weight were used for tests, whose lengths at each dosage were 3, 6, 9, and 12 mm, respectively. The traditional EICP-solidified sand was taken as a control group. After obtaining the optimal length and dosage of PVA fiber through the UCS and CCC tests, the sand strengthening mechanism by fiber-reinforced EICP technology was analyzed from a microscopic perspective using a USB handheld microscope and SEM. The specific test scheme is shown in Table 3. “A” and “B” in the test number “U-A-B” indicate the fiber length and content, respectively. With 13 groups of treatment methods and 3 parallel soil column samples in each group, a total of 39 soil column samples were prepared.

#### 2.3.1. UCS Test

The top and bottom surfaces of the prepared soil samples were polished with sandpaper. After the samples were dried, the UCS tests were conducted at a uniform loading rate of 0.5 mm/min with an automatic pressure testing machine, and the maximum axial stress of the sample was taken as UCS.

#### 2.3.2. CCC

After performing the UCS tests, approximately 30 g of material was collected from the top (t), middle (m), and bottom (b) parts of the damaged sample and crushed. The accurate mass of each piece of material (*M*_1_) was weighed. Afterward, the material was soaked in HCl (2 M) until there were no bubbles. The material was dried, and its mass (*M*_2_) was weighed again. The difference in material mass before and after acid pickling was the mass of CaCO_3_ precipitated during EICP (*M*_c_). The CCC in different parts of the soil column *C_i_* (*i* = t, m, b) was defined as the percentage of *M*_c_ to *M*_1_ (Equation (1)), and the spatial difference coefficient of the CCC (*C*_v_) was defined as the ratio of the standard deviation of CCC (*C*_s_) to the average value of CCC (*A*_v_) (Equation (2)). Both *C*_s_ and *C*_v_ were used to measure the dispersion of CCC in different parts of the fiber-reinforced EICP-solidified sand samples.

(1) $$C_{i}=M_{c}/M_{1}=\left(M_{1}-M_{2}\right)/M_{1}\times 100\%$$

(2) $$C_{v}=C_{s}/A_{v}\times 100\%$$

#### 2.3.3. Microscopic Test

The fiber lap mode and the location of CaCO_3_ precipitation in the cracks of soil samples during the strength test were observed using a USB handheld microscope; afterward, approximately 50 g of material was removed from the centre of the failure samples, and the morphology and distribution of precipitated CaCO_3_ as well as the connection between sand, fiber, and CaCO_3_ were observed via SEM.

## 3. Results

**Figure 2:**
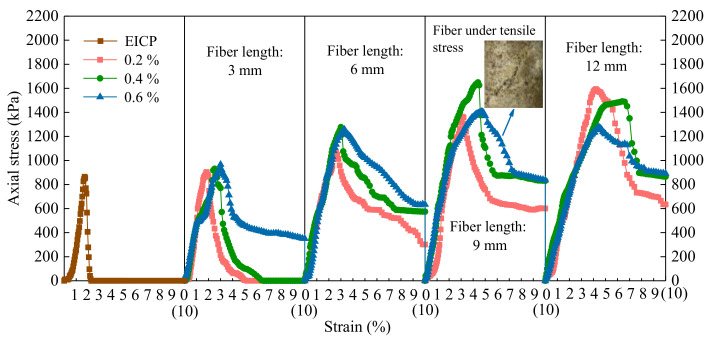
Stress-strain curves.

The UCS and CCC test results of the 13 groups of soil columns are shown in Table 4. The representative stress-strain curves selected from each group of soil samples is summarized in Figure 2. In the subsequent analysis, “Ave” and “*A*_v_” in Table 4 were taken as UCS and CCC of each group of samples, respectively.

## 4. Discussion

### 4.1. UCS

**Figure 3:**
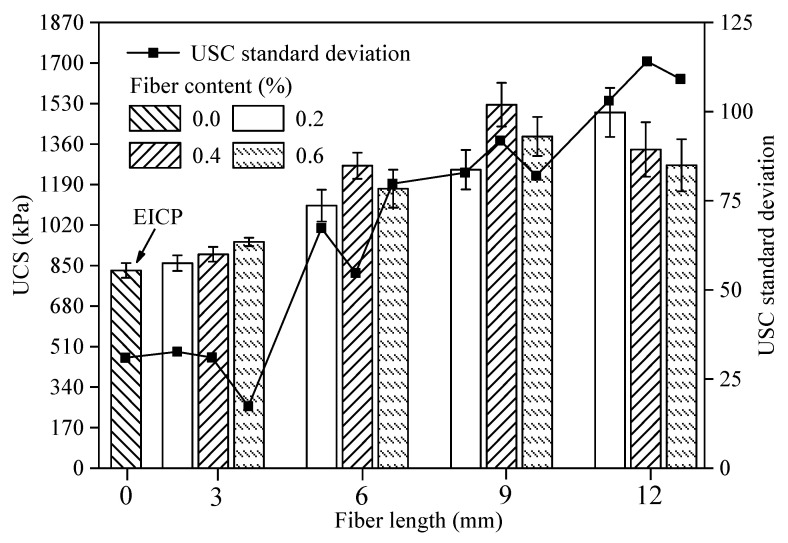
UCS and strength standard deviation of fiber-reinforced EICP-treated samples.

The UCS standard deviation was used to reflect the strength dispersion of fiber-reinforced EICP-treated sand. The value and its standard deviation of UCS for each sample after treatment are shown in Figure 3. Since the injection of EICP treatment solution in this test circulated only seven times, compared with the results of Fang et al. and Zhao et al. [20,21], the cementing efficiency and UCS herein are generally low.

Compared to the UCS of traditional EICP-treated samples, the effect of adding 3 mm fiber on UCS improvement of sands treated by EICP was considerably small, while the addition of PVA fibers with lengths of 6, 9, and 12 mm had more significant effects on the strength improvement, with the 9 mm fiber having showed the best effect. This is because the fiber length of 3 mm is short, as it is only 1.5 times the maximum particle size of sand (2 mm). The fiber had weak force transfer performance and poor reinforcement effect in soil. However, fibers that are excessively long are prone to bend, which inhibits the force transfer of fiber and affects the overall mechanical performance of the soil sample, resulting in loss of strength. A certain length of fiber can effectively fill the gaps between sand particles and play an anchoring role between CaCO_3_ and sand particles. When fibers of 3, 6, 9, and 12 mm in length were added, the fiber content corresponding to the maximum UCS was 0.6, 0.4, 0.4, and 0.2%, respectively. When the fiber length was 12 mm, UCS decreased with the increase of fiber content. Perhaps the fiber excess caused the fibers to entangle into clusters, which aggravated the uneven distribution of the fibers, resulting in a large amount of CaCO_3_ precipitation around the cluster fibers [22] and thus forming a weak reinforcement area. In fact, as fiber length increased, the probability of fiber clustering also increased, and the position of fiber clustering was the first to be destroyed, which reduced the average strength of the samples. The dispersion and non-uniformity of soil strength were increased by incorporating fiber, and the strength standard deviation enlarged with increasing fiber length.

### 4.2. Stiffness

According to Figure 2, the stress-strain curve of the samples without fiber and with small fiber length or content can be divided into two stages: rapid increase of stress and rapid decrease of stress, showing significant brittle failure characteristics. When the fiber length or content was large, the stress-strain curve of samples presented a three-stage type, which had a third stage, the residual stress stage, more than before. In the cracks where the soil column was damaged, although the tensile strength of the treated sand was 0, the mixed fiber itself could withstand a certain tensile stress, and the soil sample could still bear a certain load (residual stress strength) even if there were cracks [23,24], which inhibited the further development of cracks and prolonged the time for the overall destruction of samples.

The parameters of residual stress *σ*_r_, peak strength strain *ε*_f_, initial elastic modulus *E*, and secant modulus *E*_50_ obtained from Figure 2 are shown in Table 5. With the increase of fiber length and content, the embedded depth and filling density of fibers in samples increased gradually, the fibers overlapped each other to form a network, the bridging effect was strengthened, and the residual stress *σ*_r_ after failure increased gradually from zero. Except for sample U-12-0.6, *ε*_f_ increased with the increase of fiber length and content, and under the same fiber content; basically, the larger the fiber length, the greater the *ε*_f_ [25]. The sample U-12-0.4 had the largest *ε*_f_, which was 276.5% higher than that of treated via traditional EICP. It shows that the fiber does improve the toughness of the treated sand. For fibers with a length in the range of 3 to 9 mm, the greater the content and length of fiber, the better the soil toughness. *E* and *E*_50_ were about 1.2 to 3.9 times of that of EICP-treated soil after fiber was incorporated. The variation of *E* and *E*_50_ with the length and content of the fiber was basically consistent with the variation of strength [26]. Under the same fiber length, the fiber content corresponding to the position where the maximum stiffness appeared basically showed a decreasing trend. It can be considered that the UCS increase of fiber-reinforced EICP-treated sand originated from the increase of elastic modulus and stiffness, and the increase of residual strength and toughness was mainly due to the expansion of elastic strain range.

### 4.3. CCC and Its Spatial Distribution

**Figure 4:**
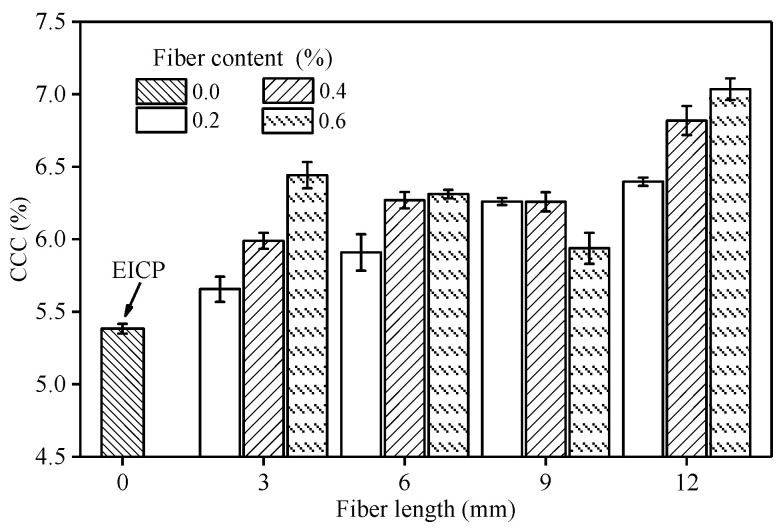
CCC of fiber reinforced EICP-treated samples.

Figure 4 shows the CCC of each sample after fiber incorporation. The CCC after adding fiber was increased by about 10–36% compared with EICP-treated soil, indicating that the addition of fiber promoted the precipitation of CaCO_3_ in EICP and increased its production. Zheng et al. [27] found that fiber has little effect on CCC when using MICP technology to solidify sand. This may be due to the fact that MICP technology has its own microbial macromolecules to provide nucleation sites. Although the urease used in EICP is free and lacks nucleation sites, the surface of the incorporated fiber can absorb a large amount of free urease, which can improve the utilization rate of urease and cementation solution and effectively make up for the lack of nucleation sites in EICP. When the fiber length was 3, 6, or 12 mm, CCC increased with the increase of fiber content; when the fiber content was 0.2 or 0.4%, CCC rose with the increase of fiber length.

Table 5 shows the growth rates of CCC and UCS (*α*_c_, *α*_s_) of sand samples strengthened with fiber-reinforced EICP compared with samples only treated with EICP, and the changes of CCC (*β*_cc_, *β*_cl_) and UCS (*β*_sc_, *β*_sl_) with another factor under the condition of same fiber length or content. The addition of fiber could increase the UCS of EICP-treated sand by up to 84%, but the highest increase of CCC was only 36%. The sample U-12-0.6 had the highest CCC, but its UCS was relatively low; while the sand sample U-9-0.4 had the highest UCS, but its CCC was only at the average level of CCC of each sample with fiber additions.

Results show that the increase of CCC is only one reason for the improvement of UCS of fiber-treated sand, and the level of CCC is not the key factor and sufficient condition to determine the UCS of fiber-reinforced EICP-treated sand [28,29,30,31]. There was no one-to-one correspondence between UCS and CCC. The variation range of *β*_cc_ and *β*_sc_ was smaller than that of *β*_cl_ and *β*_sl_, so the influence of fiber content on CCC and UCS was less than that of fiber length.

**Figure 5:**
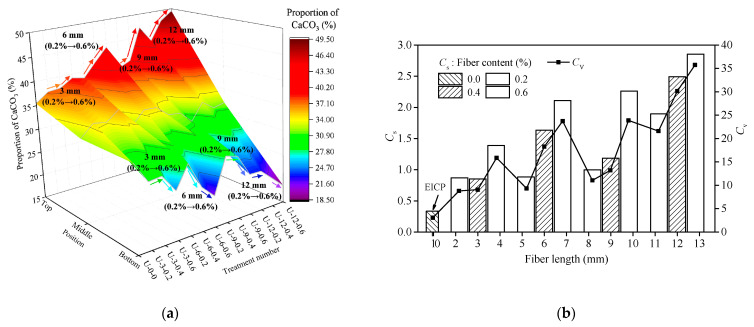
CCC of: (**a**) distribution and (**b**) coefficient of spatial difference and standard deviation.

To analyze the uniformity of CaCO_3_ distribution in the top, middle, and bottom parts of a fiber-reinforced EICP-treated sand column, the proportion of CaCO_3_ was defined as the ratio of CCC in different parts of the sand column *C_i_* (*i* = t, m, b) to the sum of CCC in the three parts. The proportion of CaCO_3_ in the top, middle, and bottom parts of the sample under different treatment methods is shown in Figure 5a. Figure 5b reflects the changes of *C*_s_ and *C*_v_ with the length and content of the added fiber. With the continuous dripping of CaCl_2_–urea mixture, CaCO_3_ crystals first precipitated at the top part of the sample, and the sand particles were cemented by the crystals, resulting in the decrease of porosity and permeability of the samples, which hindered the flow of the mixture from up to down to a certain extent, and more treatment solution remained at the top part of the sand column. The distribution rule of CaCO_3_ was top > middle > bottom, which is in accordance with Darcy’s law.

With the increase of fiber content and length, the proportion of CaCO_3_ in the top part of the samples continued to increase, the proportion of CaCO_3_ in the middle part changed slightly, and the proportion of CaCO_3_ in the bottom part continued to decrease (Figure 5a). *C*_v_ and *C*_s_ increased monotonously with the increase of fiber length or content (Figure 5b). It is noteworthy that when no fiber was added the proportion of CaCO_3_ in the top part of the samples was 1.1 times of that in the bottom part. After adding fiber, the proportion of CaCO_3_ in the top part increased to 1.3–2.7 times of that in the lower part, which shows that the non-uniformity of CaCO_3_ distribution rises with the increase of fiber length and content. This may be due to the addition of fiber, which made the top part of the sample precipitate more CaCO_3_ crystals at the same time and further increased the resistance of the treatment solution to flow downward. The uneven distribution of CaCO_3_ would form a weak area, resulting in a decrease in the integrity of soil strength after reinforcement [32], and a greater dispersion of soil strength.

### 4.4. Relationship between UCS and CCC

**Figure 6:**
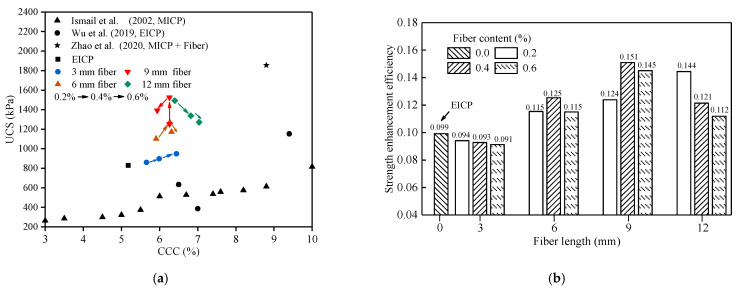
(**a**) Relationship between UCS and CCC; (**b**) strength enhancement efficiency of specimens.

Figure 6a compares the relationship between UCS and CCC in this study and other studies. The comparison data includes soil samples treated by MICP with fiber addition (Zhao et al. [21]) or without any addition (Ismail et al. [33]), and samples treated by EICP without any addition (Wu et al. [19]). The results show that the reinforcement effect of EICP-treated sand without any addition in this paper was better than that of MICP-solidified sand on the whole and it was also better than that of two fine grains of the three kinds of sand used by Wu et al. As the sand particles used by Zhao et al. were coarser than those used in this study and the total amount of treatment solution added in the test was larger, the strength of the sand column cemented by Zhao et al. through MICP combined with fiber was higher. When the fiber length was 3 mm, the relationship between UCS and CCC increased linearly with the fiber dosage, but vice versa when the fiber length was 12 mm. When the fiber length was 6 mm and 9 mm, the data point corresponding to 0.4% content was more abrupt, which made the UCS change with CCC in a two-stage folded line. The variation of UCS with CCC at different fiber lengths shows different trends, indicating that the UCS of solidified samples may be related to CCC, the spatial distribution of CaCO_3_ precipitated in the sample, the combination form of fiber–CaCO_3_–sand, and many other factors. 

The strength improvement efficiency was defined as the UCS corresponding to the unit mass of CaCO_3_ generated during the EICP process. Figure 6b compares the strength improvement efficiency of EICP-cemented samples with different fiber lengths or fiber contents. When 3 mm-long fiber was added, the tensile strength of the fiber and the bond between the fiber and the cemented sand could not be brought into full play because the fiber itself was too short, and the strength improvement efficiency was lower than that of traditional EICP-treated sand. However, the strength improvement efficiency was significantly improved by adding 6, 9, and 12 mm fibers. When adding 0.4% of 9 mm-long fiber, the strength improvement efficiency was the highest with a value of 0.151, which is 1.53 times of that of traditional EICP-solidified sand.

### 4.5. Microstructures

#### 4.5.1. USB Handheld Microscope

**Figure 7:**
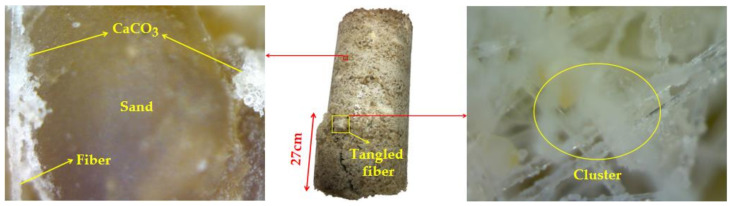
USB handheld microscope image of U-12-0.4.

Figure 7 shows the failure morphology of sample U-12-0.4 observed via USB handheld microscope after the UCS test. It is possible that the bottom part of the sample had lower CCC, so the failure often occurred in the bottom part. A large number of CaCO_3_ crystals were adsorbed on the surface of the fiber, which improved the roughness of the fiber; some crystals were bonded between the sand and the fiber, and the interfacial forces (cohesion and friction) between the fiber and the sand grain were enhanced.

When the solidified sand was subjected to external load, the fibers scattered in the sand could not only connect the evenly distributed and non-uniformly distributed parts of CaCO_3_ to form a whole through bridging action, but also act as “tie bars” to transfer stress and limit soil deformation, which effectively slowed down the development of tension cracks and improved its ductility. When the fiber length and content were excessively large, fiber clusters would appear, forming the weak area of soil reinforcement, resulting in the strength reduction.

#### 4.5.2. SEM

**Figure 8:**
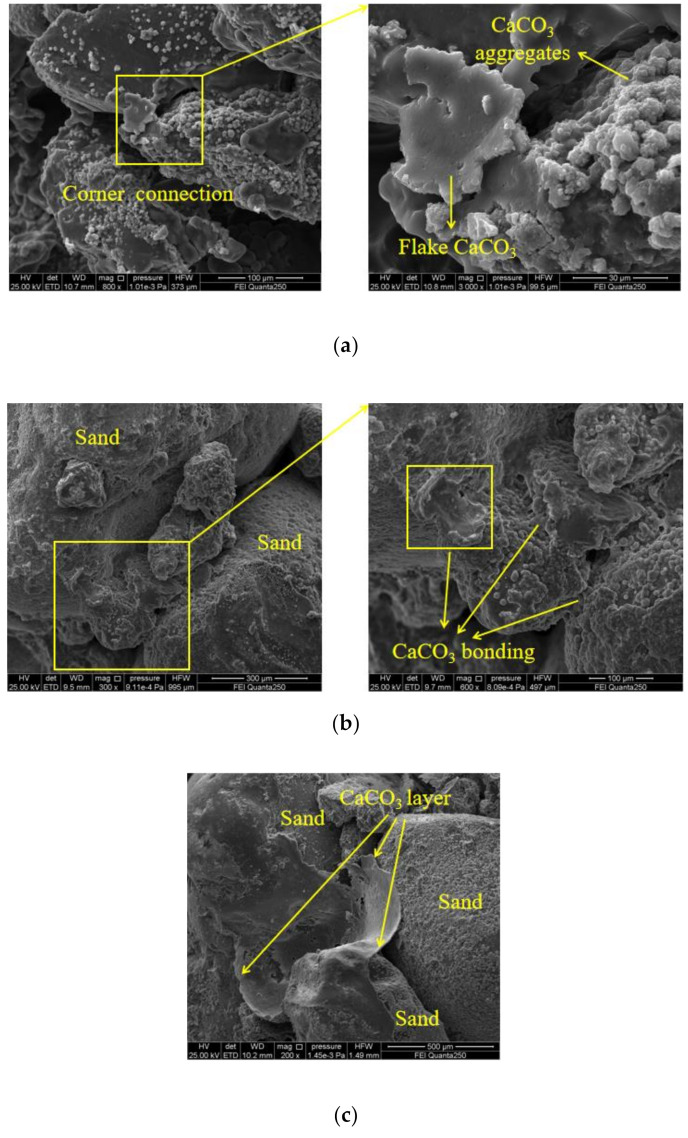
SEM images of EICP-reinforced sand column: (**a**) filling and linking; (**b**) cementation; and (**c**) encapsulation.

**Figure 9:**
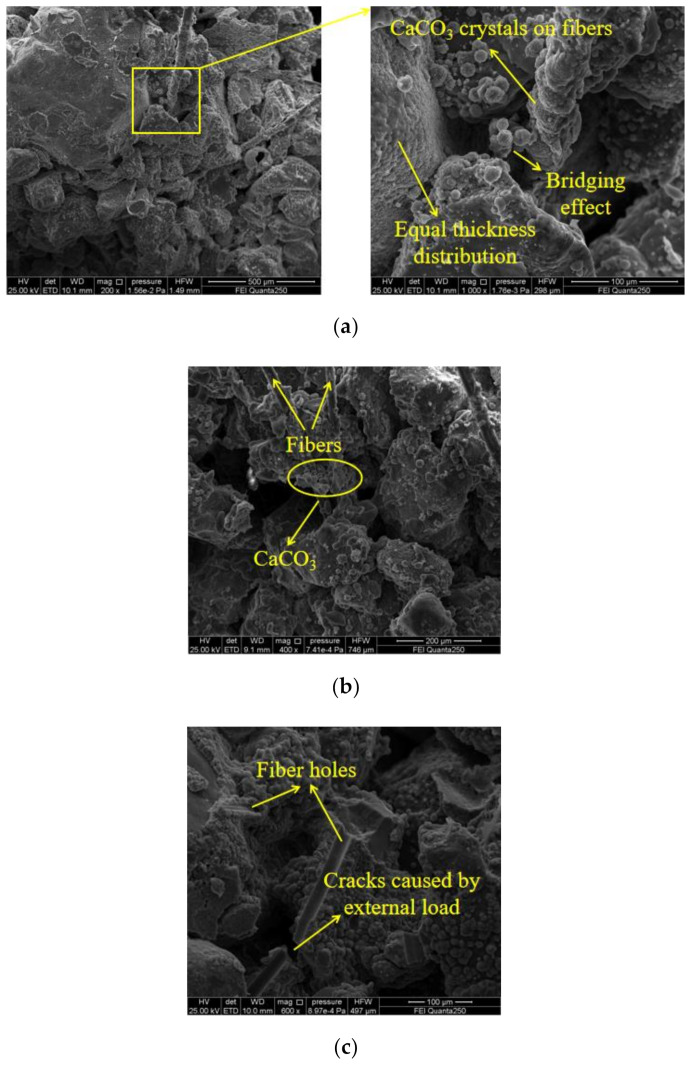
SEM images of fiber-reinforced EICP-treated sand: (**a**) fiber-filled voids and spliced sand particles; (**b**) fiber connection; and (**c**) fiber hole.

The SEM images of sand samples strengthened by EICP and fiber-reinforced EICP are compared in Figure 8 and Figure 9. Figure 8 corresponds to the three mechanisms of EICP-strengthened sand. In Figure 8a, a large amount of CaCO_3_ is scattered on the surface of sand particles, partially clustered in layers. When the soil was subjected to an external load, the CaCO_3_ surface layer with high friction strength covering the surface of sand particles would have a certain restriction on the displacement of sand particles. For some areas with large sand grain gaps, the injected CaCl_2_–urea mixture flowed to this region preferentially and stayed in this region for a longer time than other regions, so the induced reaction in this area was more sufficient and intense [34]; finally, the precipitated CaCO_3_ crystals formed a cementation layer (Figure 8b). Compared with Figure 8a, this situation was rare. The surface layer of CaCO_3_ formed in Figure 8c could wrap the adjacent sand grains and enhance the compactness and integrity of local sand grains.

According to Figure 9a, the addition of fiber filled the gaps between sand particles and made the sand denser. The decrease of sand porosity is considered the main factor for the decrease of brittleness of fiber-reinforced EICP-treated sand [35,36,37]. By absorbing a large amount of CaCO_3_ crystals, the fiber weakens the characteristic of equal thickness distribution of CaCO_3_ on the surface of sand particles [38], forming a system of “fiber–CaCO_3_–sand particles” [39]. At the same time, some pores between sand particles were filled with spherical CaCO_3_, which was due to the large amount of soybean urease solution stored between fiber and sand particles, where CaCO_3_ crystallization was regulated by organic matter. As shown in Figure 9b, the adjacent fibers were bonded by CaCO_3_ and a small amount of fibers were overlapped with each other to form a three-dimensional mesh in the sand, further enhancing the “reinforcement” effect of the fibers. Figure 9c shows the trace of fiber dislocation and the fracture of CaCO_3_ under exterior load.

## 5. Conclusions

In this study, a series of UCS, CCC, and microscopic tests on PVA fiber-reinforced EICP-treated sand specimens were performed to determine the feasibility of improving the mechanical properties of EICP-solidified soil with PVA fiber. The following conclusions can be drawn:

1. Very short-length fibers have a poor effect on the reinforcement of sand, and their effect on improving the UCS of EICP-treated sand is extremely weak. If the fiber content is excessively large, it is easy to cluster, forming a weak area of soil reinforcement, which is not conducive to the overall improvement of strength. However, the content and length range of fibers in this study can significantly improve the CCC. From the perspective of increasing the strength enhancement efficiency, the optimum fiber length and content are 9 mm and 0.4%, respectively. Fiber length has a greater effect on CCC and UCS than fiber content.

2. The addition of PVA fiber can increase the UCS of EICP-treated sand by 84%, and *E* and *E*_50_ by 20% to 290%. However, the CCC is only 36% higher than the previous maximum. The level of the CCC is not the key factor to determine the UCS of fiber reinforced EICP-treated sand; *σ*_r_ and *ε*_f_ basically increased with the rise of fiber length and content, and the maximum increase of *ε*_f_ was 276.5% compared to traditional EICP-treated sand. The UCS improvement of fiber reinforced EICP-treated sand is due to the increase of elastic modulus and stiffness, and the increase of residual strength and toughness is mainly originated from the extension of elastic strain range.

3. Microscopic images show that the CaCO_3_ deposited on the fiber surface enhances the binding force between the fiber and sand particles and forms a “fiber–CaCO_3_–sand” structure system through the bridging action of the fiber. The fibers overlap each other to form a three-dimensional mesh, which constrains the displacement and deformation of sand.

4. Incorporating fibers reduces the uniformity of EICP-solidified sand. In severe cases, the failure will develop along the path of minimum structural resistance (the least CaCO_3_ distribution), thus weakening the mechanical properties of the treated sand. Therefore, future studies can investigate the engineering properties of EICP-solidified sand with different lengths of fiber mixing. The adoption of better grouting technologies can lead to further improvement of the uniformity, strength, and toughness of fiber-stabilized sand.

## Figures and Tables

**Table 1:** Particle size distribution of Xiamen standard sand.

**Particle Size (mm)**	<2	<1	<0.5	<0.25	<0075
**Content (%)**	100.00	89.16	47.57	18.00	10.97

**Table 2:** Properties of sand and PVA fiber.

Property	Symbol	Value	Unit
	Sand		
Specific gravity	*G*_s_	2.637	—
Mean particle diameter	*d*_50_	0.29	mm
Uniformity coefficient	*C*_u_	9.62	—
Curvature coefficient	*C*_c_	2.82	—
	PVA fiber		
Diameter	*d*	20 ± 2	μm
Length	*l*	3, 6, 9, 12 ± 0.5	mm
Density	*ρ*	1.3	g cm^−3^
Tensile strength	*T*	1700	MPa
Elastic modulus	*E*	45	GPa
Dry breaking elongation	*δ*	6	%

**Table 3:** Test scheme.

Test Number	Fiber Length (mm)	Fiber Content (%)	Number of Tests			
			UCS	CCC	SEM	USB Handheld Microscope
U-0-0	0	0	3	9	1	1
U-3-0.2	3	0.2	3	9	1	1
U-3-0.4	3	0.4	3	9	1	1
U-3-0.6	3	0.6	3	9	1	1
U-6-0.2	6	0.2	3	9	1	1
U-6-0.4	6	0.4	3	9	1	1
U-6-0.6	6	0.6	3	9	1	1
U-9-0.2	9	0.2	3	9	1	1
U-9-0.4	9	0.4	3	9	1	1
U-9-0.6	9	0.6	3	9	1	1
U-12-0.2	12	0.2	3	9	1	1
U-12-0.4	12	0.4	3	9	1	1
U-12-0.6	12	0.6	3	9	1	1

**Table 4:** Test results of UCS and CCC.

Test Number	UCS (kPa)				CCC (%)			
	1	2	3	Ave	*C*_t_ ^1^	*C*_m_ ^2^	*C*_b_ ^3^	*A*_v_
U-0-0	789.36	864.81	833.793	829.32	5.505	5.011	5.017	5.178
U-3-0.2	851.72	824.95	903.64	860.1	6.331	5.87	4.767	5.656
U-3-0.4	858.71	899.68	934.63	897.67	6.743	6.132	5.093	5.989
U-3-0.6	966.43	925.44	955.72	949.19	7.563	6.974	4.79	6.442
U-6-0.2	1072.98	1037.12	1194.43	1101.51	6.843	5.709	5.176	5.909
U-6-0.4	1331.55	1277.68	1198.29	1269.17	7.797	6.5313	4.48	6.269
U-6-0.6	1065.97	1194.33	1257.75	1172.68	8.36	6.546	4.03	6.312
U-9-0.2	1204.45	1184.2	1369.33	1252.66	7.352	6.101	5.329	6.261
U-9-0.4	1474.63	1654.36	1447.25	1525.41	7.563	6.037	5.174	6.258
U-9-0.6	1405.87	1285.44	1485	1392.1	8.376	5.307	4.131	5.938
U-12-0.2	1593.81	1533.54	1351.32	1492.89	8.389	6.375	4.429	6.398
U-12-0.4	1367.94	1185.13	1459.62	1337.56	9.765	6.431	4.26	6.819
U-12-0.6	1400.65	1133.67	1279.5	1271.28	10.446	6.748	3.91	7.035

^1^ *C*_t_, ^2^ *C*_m_, and ^3^ *C*_b_ correspond to the average values of CCC in the top, middle, and bottom parts of the three soil columns in a certain group, respectively.

**Table 5:** Parameters obtained from UCS and CCC tests.

Treatment Number	*σ*_r_ (kPa)	*ε*_f_ ^1^ (%)	*E* (MPa)	*E*_50_ ^2^ (MPa)	*α*_c_ ^3^ (%)	*α*_s_ ^4^ (%)	*β*_cc_ ^5^ (%)	*β*_sc_ ^6^ (%)	*β*_cl_ ^7^ (%)	*β*_sl_ ^8^ (%)
U-0-0	0	1.7	20.10	35.06	—	—	—	—	—	—
U-3-0.2	0	1.9	23.28	46.23	9.24	3.71	—	—	—	—
U-3-0.4	0	2.5	23.81	46.72	15.68	8.24	5.89	4.37	—	—
U-3-0.6	350.25	3.0	37.06	46.71	24.43	14.45	7.56	5.74	—	—
U-6-0.2	301.45	2.5	40.38	48.90	14.13	32.82	—	—	4.48	28.07
U-6-0.4	574.30	3.0	53.29	45.32	21.09	53.04	6.09	15.22	4.68	41.38
U-6-0.6	634.38	3.3	43.73	48.59	21.91	41.40	0.68	−7.60	−2.02	23.54
U-9-0.2	600.80	3.1	24.34	44.80	20.92	51.05	—	—	5.95	13.72
U-9-0.4	831.22	4.4	79.43	53.78	20.87	83.94	−0.04	21.77	−0.18	20.19
U-9-0.6	837.12	4.7	38.80	50.04	14.68	67.86	−5.11	−8.74	−5.93	18.71
U-12-0.2	634.53	4.2	42.85	35.89	23.56	80.01	—	—	2.19	19.18
U-12-0.4	865.07	6.4	62.37	41.88	31.69	61.28	6.58	−10.40	8.96	−12.31
U-12-0.6	892.12	4.4	32.34	35.99	35.87	53.29	3.17	−4.96	18.47	−8.68

^1^ *ε*_f_ is the strain corresponding to the peak stress, ^2^ *E*_50_ is the secant modulus at 50% of the UCS, ^3^ *α*_c_ and ^4^ *α*_s_ are the increasing proportion of CCC and UCS after adding fiber compared with that of traditional EICP-treated samples. ^5^ *β*_cc_ and ^6^ *β*_sc_ represent the increased ratios of CCC and UCS higher than that at the previous fiber content, ^7^ *β*_cl_ and ^8^ *β*_sl_ represent the increased percentages of CCC and UCS higher than that of the former fiber length, respectively. Negative values in the last four columns indicate a decrease in strength.

## Data Availability

The test steps can be repeated. The data and materials obtained were true and effective. The data used to support the findings of this study are available from the corresponding author upon request.
